# Dimethyl 2-[(acridin-9-yl)methyl­idene]malonate

**DOI:** 10.1107/S1600536813000500

**Published:** 2013-01-12

**Authors:** Sinara M. V. de Almeida, Ivan R. Pitta, Maria do Carmo A. de Lima, Francisco J. B. Mendonça Junior, Carlos A. de Simone

**Affiliations:** a Laboratório de Imunopatologia Keizo Asami (LIKA), Departamento de Bioquímica, Universidade Federal de Pernambuco, 50670-901 Recife, PE, Brazil; b Laboratório de Síntese e Planejamento de Fármacos, Departamento de Antibióticos, Universidade Federal de Pernambuco, 50670-910 Recife, PE, Brazil; c Laboratório de Síntese e Vetorização de Moléculas Bioativas, Universidade Estadual da Paraíba, 58020-540 João Pessoa, PB, Brazil; d Departamento de Física e Informática, Instituto de Física de São Carlos, Universidade de São Paulo - USP, 13560-970 São Carlos, SP, Brazil

## Abstract

In the title compound, C_19_H_15_NO_4_, the acridine system is essentially planar (r.m.s. deviation = 0.015 Å). The crystal packing exhibits π–π inter­actions between pairs of centrosymmetric mol­ecules, one of them between the central heterocyclic rings and others between the outer benzene rings of the acridine systems, with centroid–centroid distances of 3.692 (1) and 3.754 (1) Å, respectively. These pairs are further linked by additional π–π inter­actions along the *a*-axis direction through one of the two outer benzene ring of neighboring mol­ecules, with a centroid–centroid distance of 3.642 (2) Å.

## Related literature

For background to acridines, see: Kumar *et al.* (2012 ▶). For the biological activity of acridine derivatives, see: Pigatto *et al.* (2011 ▶); Das *et al.* (2011 ▶); Kumar *et al.* (2012 ▶). For the synthesis of acridines, see: Tomar *et al.* (2010 ▶). For related structures, see: Buckleton & Waters (1984 ▶).
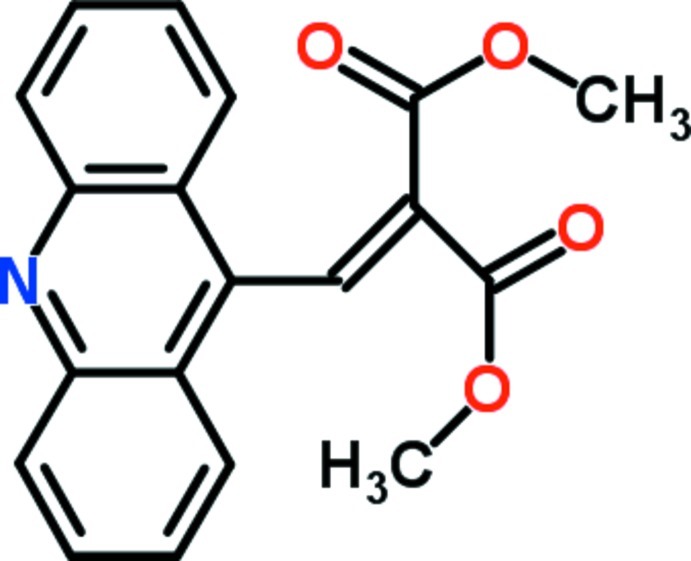


## Experimental

### 

#### Crystal data

C_19_H_15_NO_4_
*M* *_r_* = 321.32
Triclinic, 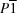

*a* = 8.3022 (2) Å
*b* = 9.0208 (3) Å
*c* = 12.0334 (4) Å
α = 96.468 (2)°
β = 93.652 (2)°
γ = 117.422 (2)°
*V* = 787.98 (4) Å^3^
*Z* = 2
Mo *K*α radiation
μ = 0.10 mm^−1^
*T* = 295 K
0.32 × 0.28 × 0.22 mm

#### Data collection

Nonius KappaCCD diffractometer
10674 measured reflections
3626 independent reflections
2805 reflections with *I* > 2σ(*I*)
*R*_int_ = 0.050

#### Refinement

*R*[*F* ^2^ > 2σ(*F* ^2^)] = 0.052
*wR*(*F* ^2^) = 0.148
*S* = 1.05
3626 reflections
218 parameters
H-atom parameters constrained
Δρ_max_ = 0.26 e Å^−3^
Δρ_min_ = −0.24 e Å^−3^

### 

Data collection: *COLLECT* (Nonius, 1997 ▶); cell refinement: *SCALEPACK* (Otwinowski & Minor, 1997 ▶); data reduction: *DENZO* (Otwinowski & Minor, 1997 ▶) and *SCALEPACK*; program(s) used to solve structure: *SHELXS97* (Sheldrick, 2008 ▶); program(s) used to refine structure: *SHELXL97* (Sheldrick, 2008 ▶); molecular graphics: *ORTEP-3 for Windows* (Farrugia, 2012 ▶); software used to prepare material for publication: *WinGX* (Farrugia, 2012 ▶).

## Supplementary Material

Crystal structure: contains datablock(s) I, global. DOI: 10.1107/S1600536813000500/lr2094sup1.cif

Structure factors: contains datablock(s) I. DOI: 10.1107/S1600536813000500/lr2094Isup2.hkl

Supplementary material file. DOI: 10.1107/S1600536813000500/lr2094Isup3.cml

Additional supplementary materials:  crystallographic information; 3D view; checkCIF report

## Figures and Tables

**Fig. 1.**
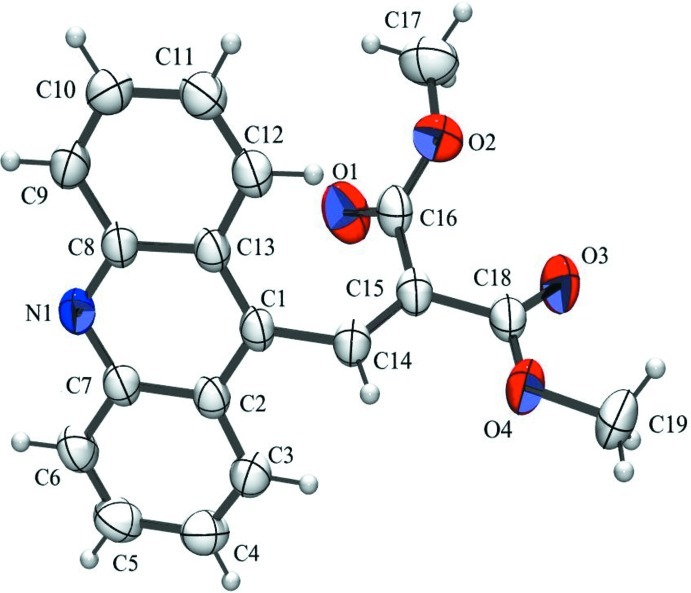
The molecular structure of the title compound. with the ellipsoids drawn at the 50% probability level.

**Fig. 2.**
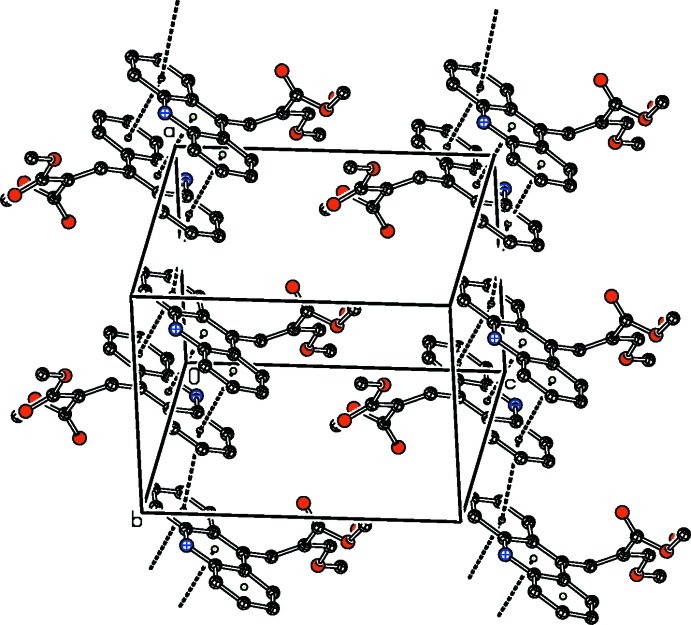
A partial view of the packing showing the π - π interactions.

## supplementary crystallographic information

### Comment

The pharmacological uses of acridine derivatives have been well documented (Kumar *et al.* 2012). Amongst these applications can be highlighted: Proflavin an antibacterial agent against many Gram positive bacteria; Bucricaine which is used topically for surface anesthesia (Kumar *et al.* 2012); Quinacrine and 9-aminoacridine that act as antimalarial and disinfectant agents respectively (Das *et al.* 2011); and several antitumor agents as nitracrina, amsacrine and 9-arylacridines which are potent topoisomerase inhibitors (Pigatto *et al.* 2011).

In this work, we report the structure of the title compound synthesized by the reaction between acridine-9-carbaldehyde and dimethyl malonate. The mean plane analysis of molecule shows that the acridine ring is essentially planar. The deviation observed is maximum for the C5 [(0.0311 (2) Å].The crystal packing exhibits π- π interactions between pairs of centrosymmetric molecules, one of them between the central heterocyclic rings and others between the side benzene rings of the acridine moieties, with centroid-centroid distances of 3.692 (1) and 3.754 (1) Årespectively. These pairs are further linked by additional π- π interactions along the *a* axis through one of the two side benzene ring of neighboring molecules, with centroid-centroid distance of 3.642 (2) Å.

### Experimental

In a Dean-Stark apparatus, acridine-9-carbaldehyde (0.1 mmol), dimethyl malonate (0.1 mmol) and morpholine (0.01 mmol) were refluxed in toluene (10 ml). The reaction mixture was refluxed at 383 K for 24 h, and the solvent was evaporated under reduced pressure. The title compound was purified by flash chromatography on silica gel (230–400 mesh) Merck (Germany), eluting with n-hexane/ethyl acetate (9.5:0.5) to give analytically pure yellow crystals of 2-acridin-9-yl-methylene-malonic acid dimethyl ester; yield 33%, *M*.p. 405–407 K. Crystals suitable for suitable for single-crystal X-ray diffraction were grown by slow evaporation at 289 K of a solution of the pure title compound in absolute ethanol.

FTIR (KBr, cm^-1^) Umax: 2954, 2924, 1730, 1628, 1435, 1266, 1232, 1076, 785, 749. NMR ^1^H (300 MHz, DMSO-*d*_6_) δ 3.15 (s, 3H), 3.92 (s, 3H), 7.65 (m, 2H), 7.89 (m, 2H), 7.99 (d, 2H,*J* = 8.6 Hz), 8.20 (d, 2H, *J* = 8.6 Hz), 8.70 (s, 1H). HRMS calcd for C_19_H_15_NO_4_ = 321.1001, found = 322.0617.

### Refinement

All H-atoms were included in the refinement at calculated positions [C—H = 0.93 Å (aromatic) and 0.96 Å (methyl), with *U*_iso_(H) = 1.2Ueq(aromatic C) or 1.5Ueq(methyl C)],also using a riding-model approximation. The maximum and minimum residual electron density peaks were located 0.16 and 0.77 Å, from the C9 and H14 atoms respectively.

### Crystal data

**Table:**

C_19_H_15_NO_4_	*Z* = 2
*M**_r_* = 321.32	*F*(000) = 336
Triclinic, *P*1	*D*_x_ = 1.354 Mg m^−^^3^
Hall symbol: -P 1	Mo *K*α radiation, λ = 0.71073 Å
*a* = 8.3022 (2) Å	Cell parameters from 6418 reflections
*b* = 9.0208 (3) Å	θ = 2.5–27.5°
*c* = 12.0334 (4) Å	µ = 0.10 mm^−^^1^
α = 96.468 (2)°	*T* = 295 K
β = 93.652 (2)°	Prism, yellow
γ = 117.422 (2)°	0.32 × 0.28 × 0.22 mm
*V* = 787.98 (4) Å^3^	

### Data collection

**Table:**

Nonius KappaCCD diffractometer	2805 reflections with *I* > 2σ(*I*)
Radiation source: Enraf Nonius FR590	*R*_int_ = 0.050
Horizonally mounted graphite crystal monochromator	θ_max_ = 27.5°, θ_min_ = 2.6°
Detector resolution: 9 pixels mm^-1^	*h* = −10→10
CCD rotation images,thick slices scans	*k* = −11→10
10674 measured reflections	*l* = −15→15
3626 independent reflections	

### Refinement

**Table:**

Refinement on *F*^2^	Secondary atom site location: difference Fourier map
Least-squares matrix: full	Hydrogen site location: inferred from neighbouring sites
*R*[*F*^2^ > 2σ(*F*^2^)] = 0.052	H-atom parameters constrained
*wR*(*F*^2^) = 0.148	*w* = 1/[σ^2^(*F*_o_^2^) + (0.0817*P*)^2^ + 0.1056*P*] where *P* = (*F*_o_^2^ + 2*F*_c_^2^)/3
*S* = 1.05	(Δ/σ)_max_ < 0.001
3626 reflections	Δρ_max_ = 0.26 e Å^−^^3^
218 parameters	Δρ_min_ = −0.24 e Å^−^^3^
0 restraints	Extinction correction: *SHELXL97* (Sheldrick, 2008), Fc^*^=kFc[1+0.001xFc^2^λ^3^/sin(2θ)]^-1/4^
Primary atom site location: structure-invariant direct methods	Extinction coefficient: 0.168 (19)

### Special details

**Table:**

Geometry. All e.s.d.'s (except the e.s.d. in the dihedral angle between two l.s. planes) are estimated using the full covariance matrix. The cell e.s.d.'s are taken into account individually in the estimation of e.s.d.'s in distances, angles and torsion angles; correlations between e.s.d.'s in cell parameters are only used when they are defined by crystal symmetry. An approximate (isotropic) treatment of cell e.s.d.'s is used for estimating e.s.d.'s involving l.s. planes.
Refinement. Refinement of *F*^2^ against ALL reflections. The weighted *R*-factor *wR* and goodness of fit *S* are based on *F*^2^, conventional *R*-factors *R* are based on *F*, with *F* set to zero for negative *F*^2^. The threshold expression of *F*^2^ > σ(*F*^2^) is used only for calculating *R*-factors(gt) *etc*. and is not relevant to the choice of reflections for refinement. *R*-factors based on *F*^2^ are statistically about twice as large as those based on *F*, and *R*- factors based on ALL data will be even larger.

### Fractional atomic coordinates and isotropic or equivalent isotropic displacement parameters (Å^2^)

**Table:**

	*x*	*y*	*z*	*U*_iso_*/*U*_eq_	
C2	0.18856 (17)	−0.04107 (16)	0.05238 (10)	0.0416 (3)	
C8	0.27051 (18)	0.29435 (17)	0.10755 (11)	0.0439 (3)	
C1	0.12960 (17)	0.00751 (16)	0.15015 (10)	0.0409 (3)	
C16	0.25132 (19)	−0.00447 (18)	0.38809 (12)	0.0475 (3)	
N1	0.32779 (16)	0.24918 (14)	0.01345 (9)	0.0477 (3)	
C14	0.02296 (18)	−0.12607 (17)	0.21649 (11)	0.0455 (3)	
H14	−0.0906	−0.2111	0.1815	0.055*	
O4	−0.19557 (15)	−0.38893 (13)	0.31911 (9)	0.0630 (3)	
C13	0.16978 (17)	0.17719 (16)	0.17992 (10)	0.0412 (3)	
C12	0.11370 (19)	0.24024 (18)	0.27615 (11)	0.0484 (3)	
H12	0.0464	0.1665	0.3236	0.058*	
C11	0.1572 (2)	0.4058 (2)	0.29913 (12)	0.0544 (4)	
H11	0.1202	0.4443	0.3625	0.065*	
C6	0.3548 (2)	0.0411 (2)	−0.11100 (12)	0.0549 (4)	
H6	0.4194	0.1228	−0.1546	0.066*	
C9	0.3139 (2)	0.46746 (18)	0.13512 (13)	0.0538 (4)	
H9	0.3808	0.5443	0.0892	0.065*	
O2	0.22592 (15)	0.05138 (14)	0.48843 (9)	0.0593 (3)	
O1	0.39648 (15)	0.04600 (18)	0.35414 (10)	0.0745 (4)	
O3	0.02856 (18)	−0.31220 (15)	0.46082 (10)	0.0716 (4)	
C7	0.28933 (18)	0.08723 (17)	−0.01311 (11)	0.0445 (3)	
C18	−0.0314 (2)	−0.28576 (17)	0.37642 (11)	0.0488 (3)	
C5	0.3247 (2)	−0.1193 (2)	−0.14145 (14)	0.0618 (4)	
H5	0.3696	−0.1466	−0.2051	0.074*	
C3	0.1592 (2)	−0.20857 (18)	0.01646 (12)	0.0508 (3)	
H3	0.0939	−0.2935	0.0577	0.061*	
C15	0.07425 (18)	−0.13590 (17)	0.32134 (11)	0.0452 (3)	
C10	0.2585 (2)	0.52130 (19)	0.22792 (13)	0.0578 (4)	
H10	0.2871	0.6345	0.2449	0.069*	
C4	0.2252 (2)	−0.2462 (2)	−0.07701 (14)	0.0588 (4)	
H4	0.2048	−0.3563	−0.0989	0.071*	
C19	−0.3052 (3)	−0.5404 (2)	0.36533 (18)	0.0765 (5)	
H19A	−0.4202	−0.6062	0.3179	0.115*	
H19B	−0.3267	−0.5095	0.4398	0.115*	
H19C	−0.2415	−0.6059	0.3690	0.115*	
C17	0.3884 (3)	0.1685 (3)	0.56385 (16)	0.0767 (5)	
H17A	0.3545	0.2006	0.6338	0.115*	
H17B	0.4557	0.2672	0.5302	0.115*	
H17C	0.4634	0.1156	0.5779	0.115*	

### Atomic displacement parameters (Å^2^)

**Table:**

	*U*^11^	*U*^22^	*U*^33^	*U*^12^	*U*^13^	*U*^23^
C2	0.0385 (6)	0.0446 (7)	0.0367 (6)	0.0149 (5)	0.0014 (5)	0.0111 (5)
C8	0.0422 (7)	0.0438 (7)	0.0379 (6)	0.0126 (5)	0.0030 (5)	0.0124 (5)
C1	0.0387 (6)	0.0446 (7)	0.0350 (6)	0.0143 (5)	0.0027 (5)	0.0143 (5)
C16	0.0493 (7)	0.0517 (7)	0.0466 (7)	0.0249 (6)	0.0081 (6)	0.0219 (6)
N1	0.0484 (6)	0.0451 (6)	0.0397 (6)	0.0120 (5)	0.0080 (5)	0.0136 (5)
C14	0.0465 (7)	0.0429 (7)	0.0428 (7)	0.0157 (5)	0.0075 (5)	0.0136 (5)
O4	0.0636 (7)	0.0495 (6)	0.0614 (7)	0.0110 (5)	0.0127 (5)	0.0236 (5)
C13	0.0395 (6)	0.0448 (7)	0.0348 (6)	0.0153 (5)	0.0023 (5)	0.0117 (5)
C12	0.0506 (7)	0.0535 (8)	0.0411 (7)	0.0230 (6)	0.0081 (6)	0.0134 (6)
C11	0.0619 (9)	0.0575 (9)	0.0440 (7)	0.0291 (7)	0.0051 (6)	0.0058 (6)
C6	0.0510 (8)	0.0602 (9)	0.0419 (7)	0.0153 (6)	0.0115 (6)	0.0105 (6)
C9	0.0574 (8)	0.0425 (7)	0.0497 (8)	0.0127 (6)	0.0043 (6)	0.0139 (6)
O2	0.0593 (6)	0.0619 (7)	0.0536 (6)	0.0279 (5)	0.0027 (5)	0.0030 (5)
O1	0.0492 (6)	0.1023 (10)	0.0627 (7)	0.0255 (6)	0.0115 (5)	0.0222 (6)
O3	0.0931 (9)	0.0618 (7)	0.0579 (7)	0.0302 (6)	0.0085 (6)	0.0315 (5)
C7	0.0399 (6)	0.0491 (7)	0.0366 (6)	0.0137 (5)	0.0038 (5)	0.0109 (5)
C18	0.0622 (8)	0.0441 (7)	0.0440 (7)	0.0252 (6)	0.0168 (6)	0.0155 (6)
C5	0.0587 (9)	0.0693 (10)	0.0482 (8)	0.0241 (8)	0.0113 (7)	−0.0008 (7)
C3	0.0500 (8)	0.0477 (7)	0.0493 (8)	0.0180 (6)	0.0048 (6)	0.0107 (6)
C15	0.0502 (7)	0.0455 (7)	0.0425 (7)	0.0220 (6)	0.0119 (6)	0.0165 (5)
C10	0.0661 (9)	0.0442 (8)	0.0545 (8)	0.0204 (7)	0.0005 (7)	0.0048 (6)
C4	0.0603 (9)	0.0542 (8)	0.0561 (9)	0.0246 (7)	0.0041 (7)	0.0001 (7)
C19	0.0826 (12)	0.0459 (9)	0.0854 (13)	0.0120 (8)	0.0267 (10)	0.0254 (8)
C17	0.0776 (12)	0.0741 (11)	0.0667 (11)	0.0323 (9)	−0.0107 (9)	−0.0063 (9)

### Geometric parameters (Å, º)

**Table:**

C2—C1	1.4037 (18)	C6—C5	1.351 (2)
C2—C3	1.425 (2)	C6—C7	1.427 (2)
C2—C7	1.4345 (17)	C6—H6	0.9300
C8—N1	1.3482 (18)	C9—C10	1.358 (2)
C8—C9	1.424 (2)	C9—H9	0.9300
C8—C13	1.4356 (17)	O2—C17	1.441 (2)
C1—C13	1.4049 (19)	O3—C18	1.1981 (17)
C1—C14	1.4823 (16)	C18—C15	1.4899 (18)
C16—O1	1.1940 (17)	C5—C4	1.415 (2)
C16—O2	1.3226 (18)	C5—H5	0.9300
C16—C15	1.497 (2)	C3—C4	1.358 (2)
N1—C7	1.3381 (18)	C3—H3	0.9300
C14—C15	1.3315 (18)	C10—H10	0.9300
C14—H14	0.9300	C4—H4	0.9300
O4—C18	1.3293 (19)	C19—H19A	0.9600
O4—C19	1.4474 (18)	C19—H19B	0.9600
C13—C12	1.4301 (19)	C19—H19C	0.9600
C12—C11	1.355 (2)	C17—H17A	0.9600
C12—H12	0.9300	C17—H17B	0.9600
C11—C10	1.419 (2)	C17—H17C	0.9600
C11—H11	0.9300		
			
C1—C2—C3	123.94 (12)	N1—C7—C6	117.91 (12)
C1—C2—C7	117.80 (12)	N1—C7—C2	123.52 (12)
C3—C2—C7	118.24 (12)	C6—C7—C2	118.55 (13)
N1—C8—C9	117.44 (12)	O3—C18—O4	124.20 (13)
N1—C8—C13	123.21 (12)	O3—C18—C15	123.33 (14)
C9—C8—C13	119.35 (12)	O4—C18—C15	112.42 (12)
C2—C1—C13	119.54 (11)	C6—C5—C4	120.40 (14)
C2—C1—C14	117.57 (12)	C6—C5—H5	119.8
C13—C1—C14	122.87 (12)	C4—C5—H5	119.8
O1—C16—O2	124.32 (15)	C4—C3—C2	121.06 (14)
O1—C16—C15	124.28 (14)	C4—C3—H3	119.5
O2—C16—C15	111.38 (11)	C2—C3—H3	119.5
C7—N1—C8	118.15 (11)	C14—C15—C18	122.41 (13)
C15—C14—C1	126.22 (12)	C14—C15—C16	122.17 (11)
C15—C14—H14	116.9	C18—C15—C16	115.18 (11)
C1—C14—H14	116.9	C9—C10—C11	120.38 (14)
C18—O4—C19	115.93 (13)	C9—C10—H10	119.8
C1—C13—C12	124.38 (12)	C11—C10—H10	119.8
C1—C13—C8	117.77 (12)	C3—C4—C5	120.61 (15)
C12—C13—C8	117.83 (12)	C3—C4—H4	119.7
C11—C12—C13	120.94 (13)	C5—C4—H4	119.7
C11—C12—H12	119.5	O4—C19—H19A	109.5
C13—C12—H12	119.5	O4—C19—H19B	109.5
C12—C11—C10	120.94 (14)	H19A—C19—H19B	109.5
C12—C11—H11	119.5	O4—C19—H19C	109.5
C10—C11—H11	119.5	H19A—C19—H19C	109.5
C5—C6—C7	121.14 (14)	H19B—C19—H19C	109.5
C5—C6—H6	119.4	O2—C17—H17A	109.5
C7—C6—H6	119.4	O2—C17—H17B	109.5
C10—C9—C8	120.55 (13)	H17A—C17—H17B	109.5
C10—C9—H9	119.7	O2—C17—H17C	109.5
C8—C9—H9	119.7	H17A—C17—H17C	109.5
C16—O2—C17	116.36 (13)	H17B—C17—H17C	109.5
